# Efficacy of Vitamin B12 and Adenosine Triphosphate in Enhancing Skin Radiance: Unveiled with a Drug–Target Interaction Deep Learning-Based Model

**DOI:** 10.3390/cimb46080537

**Published:** 2024-08-20

**Authors:** Hyeyeon Chun, Hyejin Lee, Jongwook Kim, Hyerin Yeo, Kyongeun Hyung, Dayoung Song, Moonju Kim, Seung-Hyun Jun, Nae-Gyu Kang

**Affiliations:** LG Household and Health Care R&D Center, Seoul 07795, Republic of Korea; chunhy@lghnh.com (H.C.); hellohj1223@lghnh.com (H.L.); kimjw1204@lghnh.com (J.K.); coramdeo@lghnh.com (H.Y.); hke0512@lghnh.com (K.H.); ssongdy@lghnh.com (D.S.); hoolmj@lghnh.com (M.K.); junsh@lghnh.com (S.-H.J.)

**Keywords:** skincare products, cosmetic ingredients, artificial intelligence, Vitamin B12, adenosine triphosphate

## Abstract

Skin radiance is crucial for enhancing facial attractiveness and is negatively affected by factors like hyperpigmentation and aging-related changes. Current treatments often lack comprehensive solutions for improving skin radiance. This study aimed to develop a cosmetic formula that enhances skin radiance by reducing hyperpigmentation and improving skin regeneration by targeting specific receptors—the endothelin receptor type B (EDNRB) for hyperpigmentation and the adiponectin receptor 1 (ADIPOR1) for sagging and wrinkles. To achieve this, we used artificial intelligence technologies to screen and select ingredients with an affinity for EDNRB and ADIPOR1. Vitamin B12 (VitB12) was identified as a molecule that targets EDNRB, which is involved in melanogenesis. Adenosine triphosphate (ATP) targets ADIPOR1, which is associated with skin regeneration. VitB12 successfully inhibited intracellular calcium elevation and melanogenesis induced by endothelin-1. In contrast, ATP increased the mRNA expression of collagen and elastin and promoted wound healing. Moreover, the VitB12 and ATP complex significantly increased the expression of hyaluronan synthases, which are crucial for skin hydration. Furthermore, in human participants, the application of the VitB12 and ATP complex to one-half of the face significantly improved skin radiance, elasticity, and texture. Our findings provide valuable insights for the development of skincare formulations.

## 1. Introduction

The desire to appear attractive is a universal aspiration of individuals of all ages and sexes. Skin radiance plays a crucial role in enhancing facial attractiveness and conveying a wide variety of positive impressions [1]. Studies have suggested that individuals with higher skin radiance appear younger and more attractive [1,2]. Factors contributing to the decline in skin radiance include hyperpigmentation, wrinkles, sagging, rough skin texture, and enlarged pores [2]. Therefore, we aimed to identify ingredients that can comprehensively improve these skin conditions.

Hyperpigmentation refers to the excessive synthesis and abnormal accumulation of melanin in the skin [3]. One key factor in the process of melanogenesis is the binding of endothelin-1 (ET-1) to its specific receptor, endothelin receptor type B (EDNRB) [4,5]. Elevated levels of ET-1 caused by UV irradiation of keratinocytes promote melanin synthesis [6]. Hyperpigmented lesions are characterized by enriched blood vessels and ET-1 released from endothelial cells further augments melanin synthesis [7,8]. Hyperpigmentation around vascular lesions can be attributed to EDNRB activation [7]. BQ-788, a selective EDNRB antagonist, effectively suppresses melanogenesis [5,7]. Thus, we aimed to identify EDNRB antagonists that reduce hyperpigmentation.

Facial wrinkles and sagging are common signs of skin aging and are primarily attributed to the loss of extracellular matrix components, such as collagen [9]. Adiponectin, a secreted protein derived from subcutaneous fat, promotes collagen production by dermal fibroblast [10]. Adiponectin levels decrease with skin aging [11]. Adiponectin exerts its biological effects by binding to adiponectin receptor 1 (ADIPOR1) or adiponectin receptor 2 (ADIPOR2) [12]. The expression of adiponectin and its receptor decreases with age and UV exposure, leading to skin aging [13]. Therefore, another objective was to identify ADIPOR1 agonists that enhance skin firmness and regeneration.

Over the past decade, artificial intelligence (AI) has played a crucial role in cosmetics development [14]. In a previous study, a deep-learning molecular transformer drug–target interaction (MT–DTI) model successfully predicted troxerutin as a potential TRPV1 antagonist, and subsequent experiments confirmed its efficacy in soothing skin irritation [15]. Building on this success, we leveraged the MT–DTI model to identify cosmetic ingredients with a strong binding affinity for EDRNB or ADIPOR1 with the aim of creating a cosmetic formula that enhances skin radiance. The efficacies of these ingredients were verified both in vitro and in vivo.

## 2. Materials and Methods

### 2.1. MT–DTI Model for Hit Prediction of EDNRB Antagonist and ADIPOR1 Agonist

To predict the binding affinity between the compounds and target proteins, we employed the Deargen MT–DTI paid service and adapted a modified version of a previously utilized methodology [15]. Our compound database was created by integrating and curating approximately 83,000 compounds from various sources, including ZINC15, ChemDIV, Chemspace, Molport, An FDA-approved drug library’, COCONUT, and LG Life Health’s in-house cosmetic ingredients database. All the compounds were converted into the SMILES chemical language format. To identify potential antagonist candidates for the EDNRB protein (P24350), we selected four known antagonist compounds, Bosentan, A-192621, BQ788, and IRL 2500, to train a model capable of recognizing the pharmacophore features present in the binding-site amino acid sequences. Similarly, we utilized five known agonists—adiporon, gramine, zeaxanthin, depalmitate, and arctiin—to train the model to search for potential ADIPOR1 agonist candidates.

### 2.2. Materials

Vitamin B12 (VitB12, Cyanocobalamin) was purchased from DSM Nutritional Products Ltd. (Shanghai, China), and ATP (disodium adenosine triphosphate) was purchased from Jiangsu Huayu Chemical Co. Ltd. (Nanjing, China). Endothelin-1 (ET-1), BQ-788, arbutin, gramine, and dorsomorphin were purchased from Sigma Aldrich Chemical Co. (St. Louis, MO, USA). Anti-HAS-2 antibody was obtained from Santa Cruz Biotechnology (Santa Cruz, CA, USA).

### 2.3. Cell Culture

The human melanoma MNT-1 (American Type Culture Collection (ATCC)^®^ (Manassas, VA, USA) cell number CRL-3450), the mouse melanoma B16F10 (ATCC^®^ cell number CRL-6475) cell line and the human skin fibroblast (ATCC^®^ cell number CRL-1635) were obtained from the ATCC. Immortalized human keratinocytes (HaCaT) were purchased from Addexbio (San Diego, CA, USA). MNT-1 cells were cultured in Dulbecco’s modified Eagle medium (DMEM; Solbio, Seoul, Republic of Korea) supplemented with 20% fetal bovine serum (FBS; GIBCO (Grand Island, NY, USA)), 10% AIM-V (GIBCO), 1% non-essential amino acids (NEAA; GIBCO), and penicillin-streptomycin (P/S). B16F10 and Hs68 cells were cultured in DMEM supplemented with 10% FBS and P/S. HaCaT cells were maintained in low Ca^2+^ concentration DMEM supplemented with 10% FBS and P/S. Cells were cultured at 37 °C with 5% CO_2_.

### 2.4. Intracellular Calcium Measurement

Ca^2+^ signals were measured using the Ca^2+^ indicator dye Fluo-4 (Fluo-4 Direct Calcium Assay Kit, Thermo Fisher Scientific (Waltham, MA, USA)), according to the manufacturer’s protocol. MNT-1 cells grown in a 24-well plate were incubated with Fluo-4 calcium indicator dye in a CO_2_ incubator for 30 min at 37 °C. Fluorescent images of the cells were acquired before and after reagent treatment using the EVOS FL cell imaging system (Life Technologies). Fluorescence intensity was analyzed using the freely available ImageJ 1.53e software from the National Institute of Health (NIH).

### 2.5. Melanin Content Assay

MNT-1 cells (2 × 10^5^ cells/well) were cultured in six-well plates for 24 h, followed by the addition of the treatment compounds and further culturing for 48 h. At the end of the treatment, the cells were washed in phosphate-buffered saline (PBS) and lyzed with 1N NaOH containing 10% DMSO for 10 min at 80 °C. The absorbance was measured at 400 nm using a microplate reader (Epoch, BioTek, Winooski, VT, USA). The absorbance was normalized and reported as relative melanin levels as a percentage of that of the control.

### 2.6. Isolation of Total RNA and Quantitative PCR (q-PCR)

B16F10, HaCaT, and Hs68 cells were plated in six-well plates (2 × 10^5^ cells/well). The cells were then treated with reagents for 24 h. Total RNA was extracted using an Accu-Prep^®^ Universal RNA Extraction Kit (Bioneer, Daejeon, Republic of Korea). The cDNA synthesis from total RNA was performed using AccuPower^®^ RocketScript™ Cycle (Bioneer). q-PCR was performed on an ABI7500 Real Time PCR system (Applied Biosystems) with TaqMan Gene Expression Master Mix (Thermo Fisher Scientific) and pro-inventoried TaqMan primers for MC1R (Mm00434851_s1), MITF (Mm00434954_m1), COL1A1 (Hs00164004_m1), ELN (Hs00355783_m1), HAS2 (Hs00193435_m1), HAS3 (Hs00193436_m1), and glyceralde-hyde-3-phosphate dehydrogenase (GAPDH, which was detected as a normalization control for cDNA quantity; 4352932E for a mouse or 4326317E for a human). The level of relative messenger RNA (mRNA) expression was calculated using the comparative ΔΔCt method.

### 2.7. Cell Migration Assay

Hs68 cells were plated in six-well plates at a density of 2 × 10^5^ cells/well. A single cell layer was scratched after pre-incubation at 37 °C for 24 h using a 200 μL plastic micropipette tip. The test material was then added at the indicated concentrations and incubated in a CO_2_ incubator for 24 h. Images of the wounded areas were captured using a microscope. The cell migration rate (%) was calculated by comparing the wound areas at 0 h and 24 h.

### 2.8. Immunofluorescence Analysis

HaCaT cells were plated in 24-well plates (2 × 10^5^ cells/mL) and treated with VitB12 and ATP for 48 h, followed by fixation with 4% paraformaldehyde for 10 min. The cells were then permeabilized with 0.5% Triton X-100 for 5 min. The cells were blocked with 2% BSA in PBS for 2 h at room temperature and incubated overnight with anti-HAS-2 antibodies at 4 °C. Subsequently, the cells were incubated with secondary antibodies conjugated to Fluor 488 for 1 h at room temperature. Finally, the cells were mounted in VEC-TASHIELD Antifade Mounting Medium containing DAPI. Fluorescent images were obtained using an EVOS FL Auto 2 imaging system. Relative fluorescence was quantified using Image J 1.53e software.

### 2.9. Clinical Studies

This study was conducted in accordance with the principles of the Declaration of Helsinki and was approved by the Institutional Review Board of the Korea Institute of Dermatological Sciences (Seoul, Republic of Koreas; KIDSIRB-2024-0007). A split-face comparative study was conducted at the Korea Institute of Dermatological Sciences over a period of 4 weeks (from December 2023 to January 2024). A total of 31 healthy Korean men and women aged 25–67 years (the average age was 48.13 years, with a standard deviation of 9.68.) were enrolled in the study. All participants were provided written informed consent before participation. Furthermore, the research director ensured that all participants did not meet any exclusion criteria, such as pregnancy or skin diseases. The participants applied topical toners twice daily, using an active toner containing a complex of VitB12 and ATP on one side and a placebo toner on the other side.

To evaluate skin radiance enhancement, a gloss meter (Multi Gloss 268 PLUS, Konica Minolta, Tokyo, Japan) and an imaging system (DermaView Pro, OptoBioMed Co., Gangwon, Republic of Korea) were used. The gloss meter was operated by the same examiner, and the 60° value was analyzed by conducting three repeated measurements and calculating the average value. Antera 3D (Miravex, Dublin, Ireland) was used to assess skin texture improvement. The facial images were analyzed and the average roughness (Ra) as a measure of skin texture was calculated using Antera 3D. For skin elasticity evaluation, a Cutometer MPA 580 (Courage + Khazaka Electronic GmbH, Cologne, Germany) was used. The examiner applied pressure using the probe to the cheeks of each participant and repeated the process three times. The average values of R2 (gross skin elasticity) were analyzed using its MPA CTplus software.

### 2.10. Statistical Analysis

Data are shown as means ± s.e.m. Differences between groups were analyzed using one-way analysis of variance, and corrections for multiple comparisons were made using Tukey’s multiple comparison test. Comparisons between two groups were performed using the unpaired Student’s *t*-test. Differences were considered statistically significant at *p* < 0.05.

Statistical analysis for the clinical study was conducted using SPSS software (version 17.0; IBM Corp., New York, NY, USA). The normal distribution of the measured data was tested using the Shapiro–Wilk test. A repeated-measures ANOVA (RM-ANOVA) was conducted to verify the efficacy of the applied toner. A *p* value < 0.05 was interpreted as statistically significance.

## 3. Results

### 3.1. EDNRB Antagonist and ADIPOR Agonist Prediction and Compound Selection

Using the MT–DTI model, we identified compounds that could potentially interact with EDNRB and ADIPOR1. The model identified more than 3000 compounds with potential interaction with EDNRB and approximately 2000 compounds with potential interaction with ADIPOR1. However, to ensure immediate application in cosmetics available for sale, we excluded compounds with low solubility, pigments, antibiotics, and anticancer drugs. We also excluded high molecular weight compounds that may have difficulty penetrating skin (Supplementary Materials Tables S1 and S2). To ensure safety, ingredients with a history of cosmetic use were selected. We finally selected ATP as an ADIPOR1 agonist and VitB12 as an EDNRB antagonist. ATP is known for its role as an energy source for cellular synthesis and decomposition, and VitB12 is a water-soluble vitamin that plays a crucial role in amino acid synthesis and carbon replenishment during the TCA cycle. These compounds exert beneficial effects on skin regeneration and recovery.

### 3.2. Efficacy of VitB12 as an Endothelin Receptor Antagonist

**Figure 1:**
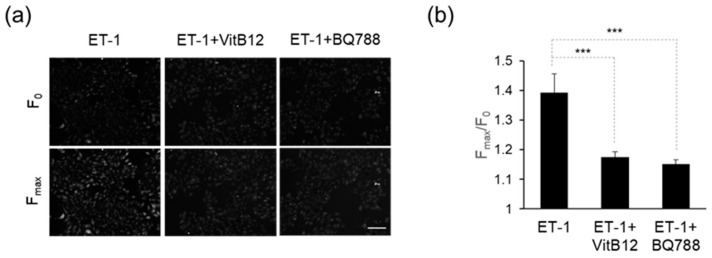
Inhibition of ET-1-evoked Ca^2+^ signals by VitB12. (**a**) Representative fluorescent images of MNT-1 cells. Images are acquired before (F_0_) and after the reagent treatment for 1 min. From each experiment, microscopy images with the maximum fluorescence signal after the reagent treatment are shown (F_max_). Scale bar = 100 μm (**b**) Bar graph showing the decrease in fluorescent intensity after VitB12 or BQ788 treatment. The fluorescence intensities were quantified using Image J. The data are shown as mean ± s.e.m. *** *p* < 0.001 by Tukey’s HSD test after the one-way ANOVA method.

We investigated the inhibitory effect of VitB12 on EDNRB expression in MNT-1 cells. ET-1 stimulates endogenous EDNRB, leading to an increase in intracellular calcium levels via phospholipase C (PLC) activation. Initially, we confirmed the elevation of intracellular calcium levels by ET-1. As shown in Figure 1a,b, the fluorescence intensity notably increased after exposure to ET-1 (F_max_) compared to that at the baseline (F_0_). The F_max_/F0 ratio for the ET-1 treatment alone was approximately 1.4. Co-treatment with ET-1 and VitB12 led to the restoration of the increased calcium levels, with an F_max_/F_0_ ratio of approximately 1.2. Similarly, co-treatment with ET-1 and BQ-788, an endothelin receptor antagonist, decreased calcium levels, with an F_max_/F_0_ ratio of approximately 1.2. These data indicate that VitB12 was effective in inhibiting stimulation-induced endothelin receptor activation by ET-1 stimulation.

**Figure 2:**
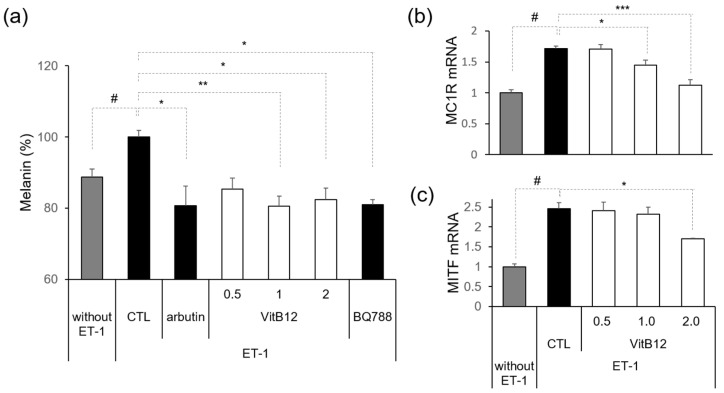
Inhibition of ET-1-induced melanogenesis by VitB12. (**a**) Melanin levels were measured after 48 h of incubation with ET-1. (**b**) Relative expression of MC1R mRNA. (**c**) Relative expression of MITF mRNA. Significance was determined by unpaired Student’s *t*-test (# *p* < 0.05) or one-way ANOVA (* *p* < 0.05, ** *p* < 0.01, *** *p* < 0.001).

We then assessed the effect of VitB12 on ET-1-induced melanin synthesis. Treatment with 10 nM ET-1 increased melanin synthesis in MNT-1 cells. Arbutin (100 μg/mL) was used as the positive control. Notably, 0.5, 1, and 2 μg/mL VitB12 significantly reduced ET-1-induced melanin synthesis, similar to the effect of BQ788 (Figure 2a). To further investigate the effects of VitB12 on melanin synthesis, we confirmed the mRNA expression of melanogenesis-related genes in B16F10 cells, melanocortin 1 receptor (MC1R), and microphthalmia-associated transcription factor (MITF). MC1R is a G-protein coupled receptor that triggers melanin synthesis, while MITF is a transcriptional factor that regulates the expression of genes encoding melanin synthesis-related enzymes. ET-1 significantly elevated the expression of MC1R and MITF. ET-1-induced MC1R expression levels were significantly decreased at 1 and 2 μg/mL (Figure 2b), and ET-1-induced MITF expression levels were significantly decreased at 2 μg/mL (Figure 2c). These data indicate that VitB12 significantly decreased ET-1-induced melanin synthesis.

### 3.3. Efficacy of ATP in Skin Regeneration

**Figure 3:**
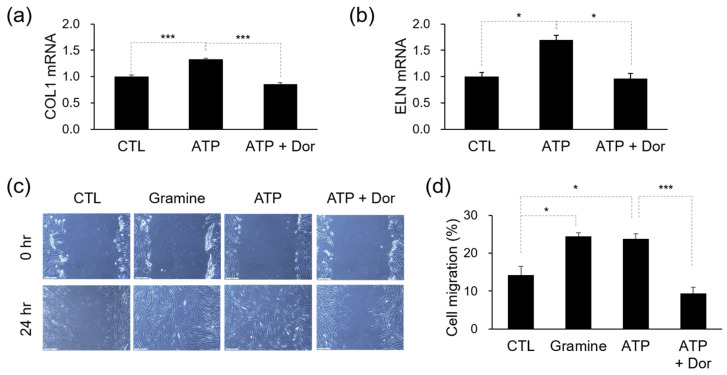
Effects of ATP on dermal fibrous component and wound healing (**a**) Relative expression of COL1 mRNA. (**b**) Relative expression of ELN mRNA. (**c**) Representative images of cell migration assay. Scale bar: 200 μm. (**d**) Bar graph showing the rate of cell migration (%) after 24 h. Data represent the mean ± s.e.m. Significance was determined by unpaired Student’s *t*-test (* *p* < 0.05, *** *p* < 0.001).

We assessed the effectiveness of ATP, which has been suggested as a potential agonist for ADIPOR1, using the MT–DTI model. The application of 10 μg/mL ATP significantly increased the mRNA levels of COL1A1, encoding collagen (Figure 3a). Additionally, 10 μg/mL ATP also led to an increase in mRNA levels of elastin (ELN), an important component that contributes to the elasticity and flexibility of the skin, along with collagen (Figure 3b). As there is currently no specific inhibitor available for ADIPOR1, we used 3 μM dorsomorphin (Dor), an inhibitor for AMP-activated protein kinase (AMPK), which is known to be downstream of ADIPOR1, to prevent the signaling of ADIPOR1 activation. Dor effectively eliminated the effects of ATP (Figure 3a,b).

Non-delayed wound healing is essential for preventing signs of skin aging, such as deep wrinkles and rough skin textures [16]. Cell migration is a critical process in wound healing [17]. Scratch wound assays were performed to study the effects of ATP on Hs68 cells. Results revealed that 10 μg/mL ATP-treated groups showed more active cell migration to the scratched area than the group without ATP treatment. Similarly, 25 μM gramine, the ADIPOR1 activator, showed similar effects to ATP. Moreover, the increased migration activity induced by ATP was attenuated by the AMPK inhibitor, 3 μM Dor (Figure 3c,d). These results indicate that ATP enhances wound healing via the ADIPOR1 pathway.

### 3.4. Increase of Hyaluronic Acid Synthases by a Complex of VitB12 and ATP

**Figure 4:**
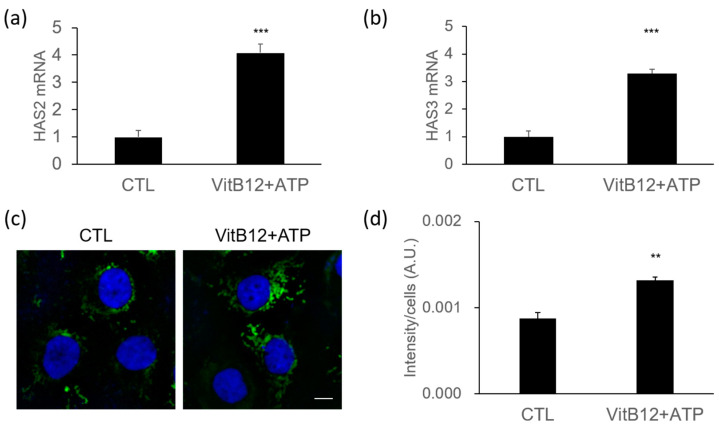
Increase in the expression of hyaluronan synthases by the VitB12 and ATP complex. (**a**) Relative expression of HAS-2 mRNA. (**b**) Relative expression of HAS-3 mRNA. (**c**) Representative images of HaCaT cells stained with anti-HAS-2 antibody (green) and co-stained with nuclear stain (DAPI, blue). Cells were treated with the VitB12 and ATP complex for 48 h. Scale bar: 10 μm (**d**) Bar graph showing the fluorescent intensity of HAS-2 after treatment with the VitB12 and ATP complex. The images were analyzed using Image J. The fluorescence intensity is expressed in arbitrary units (A.U.). Data represent mean ± s.e.m., with n = 7 samples. Significance was determined by unpaired Student’s *t*-test (** *p* < 0.01, *** *p* < 0.001).

Skin hydration is a crucial factor contributing to skin radiance [18]. We investigated the effect of the VitB12 and ATP complex on the expression of genes related to skin moisturization. Hyaluronan synthases (HAS) are enzymes responsible for producing hyaluronan (hyaluronic acid, HA), the key molecule involved in skin moisture retention. HA is associated with water retention, playing a crucial role in maintaining skin hydration [19]. There are three types of HAS: HAS-1, HAS-2, and HAS-3. To evaluate the moisturizing effects of the VitB12 and ATP complex, we measured the mRNA expression levels of HAS-2 and HAS-3 in HaCaT cells using real-time PCR. As shown in Figure 4a,b, the VitB12 and ATP complex significantly increased the mRNA expression levels of both HAS-2 and HAS-3 (*p* < 0.001).

Consistent with the mRNA expression results, treatment with the VitB12 and ATP complex also led to elevated protein expression of HAS-2 in HaCaT cells. We performed immunofluorescence analysis after a 48 h treatment and observed that the VitB12 and ATP complex significantly up-regulated the protein expression of HAS-2 compared to untreated cells (Figure 4c). The fluorescence intensity per cell increased approximately 1.44-fold (Figure 4d).

In summary, our findings suggest that the VitB12 and ATP complex effectively enhances the expression of HAS-2 and HAS-3 genes, leading to increased HAS-2 protein levels. This suggests that the VitB12 and ATP complex promotes skin hydration and may have positive effects on skin moisturization.

### 3.5. Skin Enhancement by the VitB12 and ATP Complex

**Figure 5:**
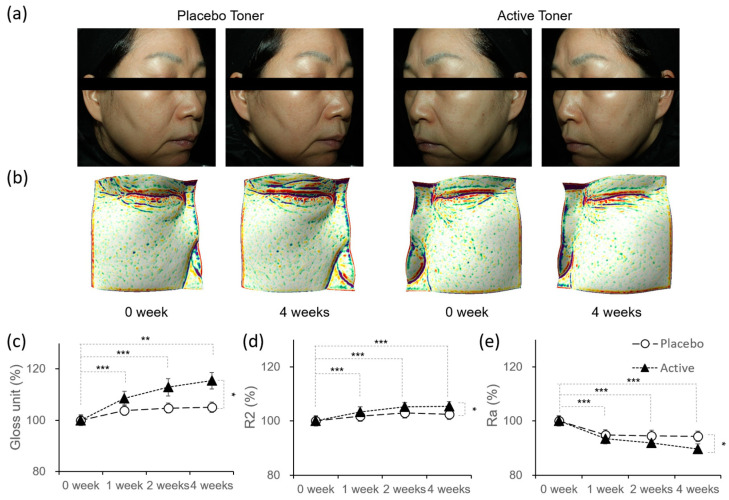
Impact of the active toner on skin radiance, elasticity, and texture. (**a**) Facial images of Derma-View before (0 weeks) and after (4 weeks) treatment with placebo toner (right side) and active toner (left side). (**b**) Facial images using the texture small filter from the Antera3D. (**c**) Change in radiance (gloss unit, %) measured by Glossmeter (**d**) Changes in elasticity (R2, %) measured by Cutometer. (**e**) Changes in texture (Ra, %) analyzed by Antera3D. The measured values, gloss unit (G.U.), R2 (%), and Ra (A.U.), are normalized to the average value at 0 weeks. Data represent means ± s.e.m. Statistical analysis was conducted using repeated measures ANOVA (* *p* < 0.05, ** *p* < 0.01, *** *p* < 0.001).

We evaluated whether the VitB12 and ATP complex improves radiance, elasticity, and texture in human skin by having 31 healthy men and women who applied an active toner containing the complex to one half of their faces and a placebo toner to the other half, twice daily for 4 weeks. To assess skin radiance, we used DermaView Pro(OptoBioMed Co., Gangwon, Republic of Korea) to acquire facial images and Multi Gloss 268 PLUS(Konica Minolta, Tokyo, Japan) to measure gloss values. Figure 5a shows a comparison of the facial skin radiance before and after using the active toner on one side and the placebo toner on the other side for 4 weeks. As shown in Figure 5c, skin gloss significantly increased at 1, 2 (*p* < 0.001), and 4 weeks (*p* < 0.01) after using the active toner. Additionally, compared with the placebo toner-treated area, the active toner-treated area exhibited significantly higher gloss values over time (*p* < 0.05). Skin elasticity was assessed by analyzing the R2 parameter measured using a Dual MPA 580 Cutometer(Courage + Khazaka Electronic GmbH, Cologne, Germany). Skin elasticity improved significantly after 1, 2, and 4 weeks (*p* < 0.001) after using the active toner compared with before use (Figure 5d). A significant difference in skin elasticity was also observed between the active and placebo toner use over time (*p* < 0.05). Skin texture was evaluated using Antera3D(Miravex, Dublin, Ireland). The images taken before and after the toner application were overlapped and analyzed using Antera3D CS ver. 3.0.2 software. A small texture filter was used to analyze the Ra value, which represents the average roughness of the skin. Figure 5b,e show a significant decrease in skin roughness in the active toner-treated area at 1, 2, and 4 weeks compared to before use. The Ra values of the active-toner-treated areas were also significantly lower than those of the placebo-treated areas over time. The VitB12 and ATP complex is blended into the active toner with dexpanthenol and glycerin to improve viscosity and stabilization, but concentrations below those that could affect the skin were used [20]. Based on these findings, we conclude that VitB12 and ATP contribute to improvements in skin radiance, elasticity, and texture.

## 4. Discussion

In the current study, we have successfully identified ingredients that have the potential to improve skin radiance by targeting specific conditions such as hyperpigmentation and sagging. The use of AI technologies, particularly the deep learning MT–DTI model, allowed us to efficiently screen and select cosmetic ingredients with a strong binding affinity to EDNRB and ADIPOR1 receptors.

Traditional molecular docking and similarity-based models have limitations in obtaining 3D structures, scaling to large datasets, and predicting new molecules, which highlights the strengths of deep learning-based models [21]. Deep learning models like DeepDTA, a convolutional neural network (CNN)-based model, can automatically extract useful features from molecular and protein sequences without manual feature engineering. However, CNNs have limitations in modeling long-range relationships between atoms, which led to using the MT–DTI model with self-attention mechanisms to overcome this [21]. This model employs self-attention mechanisms to capture all relationships within the sequence. Additionally, pre-training on molecular representations using the PubChem database allows for the learning of chemical structures and improving DTI prediction performance.

Our findings have demonstrated that VitB12 acts as an antagonist of EDNRB, effectively reducing ET-1-induced hyperpigmentation. To the best of our knowledge, this finding is novel in that VitB12 has not previously been shown to inhibit EDNRB. This finding is consistent with previous knowledge that VitB12 deficiency can contribute to hyperpigmentation in areas like fingers, knuckles, and toes [22,23]. Additionally, VitB12, also known as an NO scavenger, is commonly used in the treatment of atopic dermatitis [24]. In this study, we showed that VitB12 acts as an essential extracellular messenger and an inhibitor of EDNRB, which is believed to contribute to the alleviation of symptoms such as erythema, edema, and itching in atopic dermatitis.

ATP is an essential nucleotide found in both the intracellular and extracellular compartments [25]. Intracellular ATP functions as an energy source for various metabolic reactions [26]. In contrast, extracellular ATP serves as a signaling molecule with diverse functions, such as inflammation, cell migration, differentiation, and proliferation, depending on the cell type or activated receptors [27]. Extracellular ATP is sensed by purinergic receptors such as P2Y or P2X and plays a role in promoting skin wound healing [28]. However, inhibition of wound healing by reducing keratinocyte migration through P2Y2 activation is controversial [29]. In this study, we proposed that ATP acts as an agonist of ADIPOR1 and demonstrated its ability to facilitate wound healing in fibroblasts.

Although further validation using cellular gene editing techniques is needed to unveil the specific roles of EDNRB and ADIPOR1 in these processes, our study establishes the positive effect of the VitB12 and ATP complex on improving skin radiance, elasticity, and texture. Future research sould focus on exploring the molecular mechanisms underlying the interactions between these ingredients and their respective receptors.

## Data Availability

The data supporting this study’s findings are available from the corresponding author upon request.

## Supplementary Materials

The following supporting information can be downloaded at: https://www.mdpi.com/article/10.3390/cimb46080537/s1, Table S1: Candidate compounds predicted as EDNRB antagonists; Table S2: Candidate compounds predicted as ADIPOR1 agonists
