# Supplementary material for: Efficacy of Vitamin B12 and Adenosine Triphosphate in Enhancing Skin Radiance: Unveiled with a Drug–Target Interaction Deep Learning-Based Model
Source: Curr Issues Mol Biol. 2024 Aug 20;46(8):9082–92. doi: 10.3390/cimb46080537 (PMC11352743; doi:10.3390/cimb46080537)
Supplement: Supplementary file 1 [file cimb-46-00537-s001.zip › cimb-3138728-supplementary.pdf]

Table S1. Candidate compounds predicted as EDNRB antagonists

Name	Molecular Weight (g/mol)	predicted Kd (nM)	predicted IC50 (nM)
Ancovenin	1961.24	2.143	25.296
Tiotropium	720.962	2.339	48.925
Atogepant	603.523	3.016	46.762
Sincalide	720.962	3.743	50.641
Oscillamide C	957.143	4.228	179.945
Odevixibat	740.945	4.237	37.26
Ceruletide	1352.426	5.281	228.24
Corticiamide A	1897.769	5.828	81.497
Cyanocobalamin	1355.388	6.452	247.038
Hydroxocobalamin	1346.377	6.937	403.371
Mecobalamin	1344.405	6.975	323.343
Pasireotide	1047.227	10.02	267.804
Lypressin	1056.239	11.053	44.885
Conivaptan	498.586	11.238	248.303
Clidinium	352.454	12.703	191.041
Eluxadoline	569.662	13.521	108.883
Vorapaxar	720.962	15.019	141.952
Tamsulosin	720.962	15.135	245.672
Elagolix	631.598	17.077	102.671
Lasofoxifene	413.561	17.581	161.784
Vasopressin	720.962	17.721	60.401
Sh-oligopeptide-74	720.962	18.89	418.74
Aclidinium	484.663	21.687	133.779
Berotralstat	562.571	30.704	144.741
Netarsudil	453.542	35.802	298.467
Ubrogepant	720.962	38.744	162.595
Ilomastat	388.468	41.714	60.914
Dabigatran etexilate	627.746	49.315	235.439

Table S2. Candidate compounds predicted as ADIPOR1 agonists

Name	Molecular Weight (g/mol)	predicted Kd (nM)	predicted IC50 (nM)
Thiamine diphosphate	460.773	23.187	99.651
Ilomastat	388.468	25.578	51.254
Cocarboxylase	424.312	52.715	186.837
Aceroside Iv	458.507	71.144	261.787
Mulberroside C	458.463	130.006	310.991
Aloesinol	556.564	131.153	373.582
Apigenin 7-O-Beta-D-(6-O-P-Coumaroyl)Glucopyranoside	578.526	136.138	312.801
Rivulobirin C	590.581	138.899	88.354
Chaboside	556.524	139.349	282.635
Biondnoid A	594.525	146.365	386.238
Dracotanoside C	567.683	158.702	361.171
Aceroside B1	460.523	167.773	333.092
Aceroside I	460.523	174.687	317.974
Echinacin	578.526	191.358	372.04
Itoside C	544.509	199.347	350.3
Nyssoside	490.373	206.306	243.595
4-Hydroxytremulacin	544.509	220.62	253.883
Onoside	476.434	222.413	259.235
Luteolin 3'-Methyl Ether 7-(6''-Crotonylglucoside)	530.482	223.82	435.471
Itoside D	544.509	224.701	224.638
Levomefolic acid	459.463	227.171	150.857
Benfotiamine	466.456	237.847	482.639
Cochinchiside B	544.509	238.835	227.407
Formononetin 7-O-(2''-P-Hydroxybenzoylglucoside)	550.516	239.672	349.256
Disodium adenosine triphosphate	551.146	242.246	203.304
Tributylcresylbutane	544.82	244.393	199.844
Naucleofficine C	484.505	252.405	408.438
Methylbutylphenyl decyloxybenzoate	424.625	252.523	451.688
Isoaloeresin D	556.564	262.463	148.482
Pabularin B	574.535	264.184	105.845
Methylbutylphenyl dodecyloxybenzoate	452.679	269.979	379.198
Symplocoside	466.439	272.639	474.022
Schoenoside	434.397	277.866	316.819
Aloe C-Glucosylchromone	540.565	280.952	255.497
Bumetrizole	315.804	289.054	440.15
Kunzeachromone C	506.416	298.264	262.427

Benzethonium chloride	448.091	403.833	468.736
Methylbutylphenyl octyloxybenzoate	396.571	444.567	270.959
Pergolide	314.498	478.135	20.757
